# A Comprehensive Study of Event Detection in WPCN Networks with Noisy Measurements

**DOI:** 10.3390/s22062163

**Published:** 2022-03-10

**Authors:** Michael Koutsioumpos, Evangelos Zervas, Efstathios Hadjiefthymiades, Lazaros Merakos

**Affiliations:** 1 Department of Informatics and Telecommunications, National and Kapodistrian University of Athens, Panepistimioupolis, Ilissia, 15772 Athens, Greece; shadj@di.uoa.gr (E.H.); merakos@di.uoa.gr (L.M.); 2 Department of Electrical and Electronics Engineering, University of West Attica, 12244 Egaleo, Greece; ezervas@uniwa.gr

**Keywords:** WPCN, optimal stopping, mmWave sensor network

## Abstract

Various aspects of the detection of events in wireless powered communication networks (WPCN) are studied and analyzed under the assumption of highly noisy sensor measurements. In WPCN, networks sensor nodes’ stored energy is a scarce resource and must be treated sparingly. Frequent false alarm detections force superfluous transmissions, thus depleting nodes’ energy storage. This has an adverse effect on the probability of successful transmission of the information message and its delay in case of a true positive detection. In this work, the detection problem is approached using an optimal stopping framework, where the involved likelihoods are highly unstable due to the noisy measurements. A classical AR filter is adopted in order to smooth the posterior likelihoods prior to their usage in the detection phase and its performance is contrasted to that of a novel Beta Particle Filter smoother. The effects of the smoothing filters on the achieved false alarm rate and detection delay are examined using numerical and simulation results. Moreover, the assessment of the detection process takes into account critical WPCN parameters, such as the charging efficiency and the location of the sensors, thus aiding the system design.

## 1. Introduction

In recent years, technological advancements have paved the way for applications of wireless sensor networks in many aspects of daily life, such as environmental monitoring, agricultural monitoring, weather forecasting, and fire detection [1,2]. A wireless sensor network consists of battery-powered sensor devices dedicated to collecting information by continuously monitoring the physical environment and detecting critical events of interest. In case of a critical event, the sensor must be capable of immediately detecting and informing the network of the current situation.

A known problem for sensor nodes powered by batteries is that once the battery of a sensor is low charged or exhausted, the device becomes unavailable. Consequently, parts of the monitored area, with time, become unsupervised, affecting the performance of the entire network. It is conceivable that a network of battery-powered sensors is likely to degrade with time and special provisions should be made for its uninterrupted operation. Using wireless power transfer (WPT) technologies in such networks offers an important additional tool for extending the network lifetime by periodically charging the sensor batteries. Millimeter-wave radiofrequency technology supports high gains and is well fitted to dense access networks. As a result, using a millimeter-band antenna that conveys energy and information via the downlink is an excellent choice for maintaining the sensor network’s performance.

We focus on an event detection application using a wireless powered communication network (WPCN) under the assumption of extremely noisy measurements. To this end, we consider multiple sensor nodes scattered randomly in the monitored area that are capable of harvesting energy wirelessly. An access point (AP), equipped with a mmWave antenna, orchestrates the information exchange with the nodes and the charging process. Each sensor takes measurements from the environment and tries to detect a possible change in the distribution of the monitored parameter. In case of an event, the sensor transmits the available information at the time to the AP with as much energy as possible. As sensor measurements may be noisy due to the nature of the monitored phenomenon or the quality of the sensors themselves, the detection process may result in an extremely large number of false alarms. The sensor nodes treat each alarm as a true event and try to pass relative information to the AP. This in turn exhausts frequently the node’s stored energy since increasing the probability of successful message reception demands transmissions using all the available power. Harvesting energy from scratch regularly (especially with a small charging efficiency index) leads to partially charged nodes at any time, thus jeopardizing the functionality of the system. The aim of this work is to reduce the number of false alarms, which deplete the node energy (because of multiple unnecessary transmissions), and at the same time reduce the delay of detecting the critical event. We base the solution under investigation on Shiryayev’s theory of optimal stopping and specifically on the theory of the optimization problem of disruption [3,4].

Showing that the measurements of the posterior probability of the event occurring before the event may be quite unstable, we propose a solution to remedy the problem. A novel smoothing technique based on particle filtering principles is proposed and compared with the classic AR filtering technique.

Specifically, the main contributions of this paper are the following:

- We derive analytical expressions for the average harvested energy per slot and the probability of successful information reception for a node.
- We define the optimal stopping problem and show that the node has to postpone its transmission, at least until the accumulated energy satisfies a specific criterion.
- We propose two solutions to overcome the posterior probability measurement variability problem. The first relies on the use of an AR filter, whereas the second uses a novel technique based on the particle filter theory.
- We assess the performance of the proposed solutions through simulations.

We organize the rest of this paper as follows. Section 2 summarizes the existing related research work. In Section 3, we present the system model and provide analytical expressions of the average harvesting energy per time slot and the probability of successful information reception. In Section 4, we define and solve the optimization problem and introduce two posterior smoothing techniques, a classical AR filter and a second that relies on particle filter principles. Simulation results are presented in Section 5. Finally, Section 6 contains our conclusions.

## 2. Related Work

The feasibility of a wireless power sensor network powered by a mmWave MIMO antenna is present in [5,6]. The authors present the benefits in terms of energy harvesting efficiency. Optimal section selection algorithms are introduced and analyzed to maximize the power coverage of the network. The work in [7] considers the performance of the network in terms of energy harvest efficiency and the ability of the sensor network to successfully detect environmental changes. The authors propose a solution that balances the two criteria. The authors in [8] introduce a reporting scheme that allows sensor nodes to inform the AP about their energy levels. Based on these reports, efficient energy-beamforming strategies are devised so that, acting proactively, all sensors are sufficiently charged most of the time and are capable of successfully transmitting their information upon detection of an event.

Event detection is one of the most important tasks of sensor networks, with a wide range of applications like intrusion detection, environmental monitoring, fire detection, and outlier detection in sensor networks [9,10,11]. Event detection most of the time is expressed as a change in the distribution of a monitored parameter from the sensor network. Observations are assumed to be taken sequentially in time, and the objective is to detect the change as quickly as possible subject to the probability of false alarm. The problem of event detection in a multiple sensors network has been studied in various contexts. One area of interest is how the event is perceived from the sensor network. Works in [12,13] assume that only one sensor senses the change in the environment and its identity is unknown. An extension in [14] assumes that an unknown subset of affected sensors detects the event at the same time. The case where the sensors perceive the change at different times is studied in [15,16].

Another aspect of multiple sensor detection focuses on where the event information from the sensors is located for decision making. For the decentralized version, each sensor based on his measurements decides if a change in the monitored environment consists of an alarm [4,17]. On the contrary, for the centralized scenario [18], event information is available at several wireless sensors in the network, which transmit their information to a fusion center. The fusion center is responsible to raise an alarm as soon as possible subject to a false alarm rate constraint. The work in [19] considers the scenario where all sensors communicate their information to a fusion center. The fusion center is responsible for the decision process by monitoring the posterior probability of the received measurements. Additionally, the fusion center, in order to minimize the energy consumption of the sensors, chooses each time which sensors to keep enabled. Sensors are collocated and events happen at a random time following a geometric distribution. The proposed algorithm accomplishes to detect the intrusion (event) as early as possible using a minimal number of observations subject to a false alarm probability. One disadvantage of the method, as is concluded from the simulation results, is the computational complexity of the algorithm that depends on the number of active sensors. The authors in [20] propose a two-stage detection process in a sensor network used for fire detection. At first, each sensor based on its measurements monitors for any differences in parameter distribution. By defining a soft threshold and using a modified CUMSUM algorithm to sequentially detect the change, the sensor upon detection sends all the available information to a fusion center. At the second step, the fusion center, based on the measurements received by detecting nodes, applies a hard threshold to raise a fire alarm.

There are two common approaches to solve a change detection problem. Firstly, the minimax method [21], where the objective is to minimize the worst-case delay subject to a lower bound in the meantime between false alarms. Secondly, the Bayesian approach [3], where the time of the change (event) is assumed to be a random variable with known distribution and the goal is to minimize the detection delay subject to the probability of false alarm. Minimax optimal solution minimizes the cost under the worst-case change-time distribution, which is generally unknown. Therefore, we follow the Bayesian approach which uses prior information about the distribution of the change time. This prior distribution can be estimated using historic data. An additional constraint has been incorporated in the optimization process to accommodate the probability of successful information transmission.

## 3. System Model and Analysis

We consider a mmWave WPCN with sensor nodes scattered randomly in a circular area of radius ρ. The sensor nodes monitor the area for possible events, and upon detection of an event, they report it to the AP.

### 3.1. Network Topology

**Figure 1:**
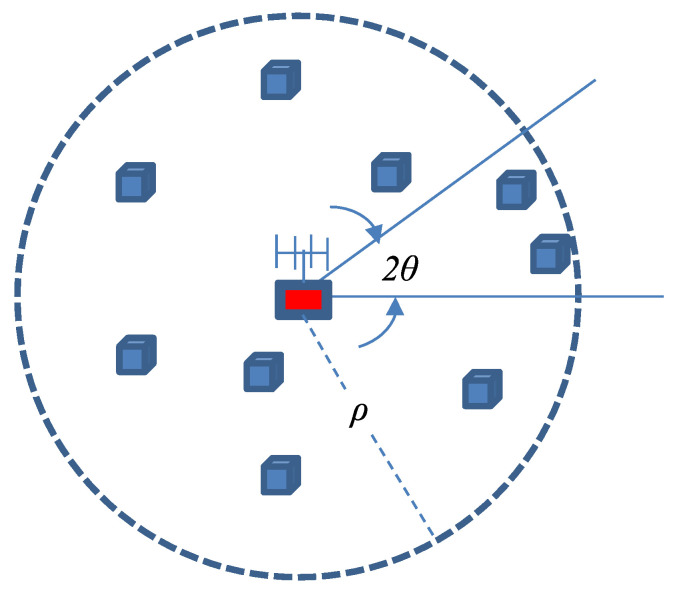
Network topology with sensor nodes depicted with boxes.

Sensor nodes are wirelessly charged by an Access Point (AP) centered at the origin of the monitored area. A uniform linear array of *M* antennas at the AP is used to switch the radiation beam along *S* sectors. Switching among sectors can be performed in a circular fashion or randomly. Figure 1 depicts the topology of the system. For this work, no blockage effect is considered but the channel between the AP and a sensor node is modeled as a fast-fading Rayleigh channel, independent for each sensor node.

**Figure 2:**
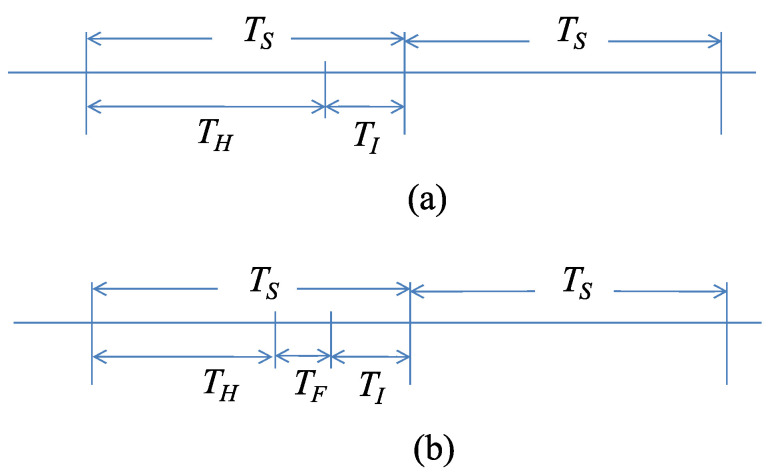
System time is slotted with slot duration $T_{s}$ equal to the residence time of the charging beam to each sector. (**a**) During each slot nodes in the served sector charge for a period $T_{H}$ and transmit (if necessary) for a period $T_{I}$. (**b**) An alternative slot format allows sensor nodes to exchange information for fusion or message relaying using period $T_{F}$.

Time is slotted and the duration of each slot, $T_{S}$, is taken equal to the residence time of the AP beam to each sector. For the time slot per se we consider two formats. In the simplest one, each slot is comprised of two fields: the harvesting period $T_{H}$ and the information period $T_{I}$, as it is shown in Figure 2a.

During the energy harvesting period ($T_{H}$), sensors located in the served sector harvest energy and charge their battery. This process is discussed in the next subsection. Upon detection of an event, the node will transmit its identity (ID) and proper information (measurements) regarding the event during the information period ($T_{I}$). To maximize the probability of successful reception of the information message by the AP, the sensor nodes use all their stored energy for transmission. Since, neighboring nodes can simultaneously detect the event, to avoid collisions of multiple transmissions at the AP, an orthogonal multiple access scheme, like FDMA, is adopted. The justification for such a choice is the abundance of bandwidth in the mmWave band and the low rate of information transmission.

The second time slot format was proposed in [8] to facilitate sensors’ feedback to the AP. This was achieved by introducing an additional field, called the reporting period, during which the sensors report their energy levels to the AP. The same time slot format, which is depicted in Figure 2b, can be used to accommodate sensor fusion. In this case, each slot is divided into three periods: the energy harvesting period ($T_{H}$), the fusion period ($T_{F}$), and the information period ($T_{I}$). $T_{F}$ is introduced to allow neighboring sensor nodes to exchange information such as their measurements, for fusion purposes, or their energy levels if more sophisticated energy-conserving schemes are desirable. Although several comments in this work refer to the second time slot format, an in-depth exploitation of its advantages is left for future work.

### 3.2. Energy Harvesting Phase

The amount of harvested energy by a node depends on the received RF power, denoted by $P^{RF}$, and the exposure time to radiation. For this work, we adopt the nonlinear model presented in [22], according to which

(1) $$E=\frac{t\gamma E_{b}\left(1-\exp\left(-\zeta_{1}P^{RF}\right)\right)}{1+\exp(-\zeta_{1}\left(P^{RF}-\zeta_{2}\right)}$$

The constants $\zeta_{1}$, $\zeta_{2}$ are circuit implementation dependent, $E_{b}$ denotes the batteries’ maximum energy storage, and the critical parameter γ∈[0,1] models the efficiency of the charging process. The harvested energy is an increasing function of the received power, $E=h(P^{RF})$, and therefore:

(2) $$P\left\{E\leq \epsilon \right\}=P\{P^{RF}\leq h^{-1}\left(\epsilon \right)\}$$

Solving Equation (1) for $P^{RF}$, we obtain

(3) $$P^{RF}=-\frac{1}{\zeta_{1}}\ln\left(\frac{t\gamma E_{b}-\epsilon }{t\gamma E_{b}+\epsilon \exp\left(\zeta_{1}\zeta_{2}\right)}\right)=\eta \left(t,\epsilon \right)$$

Thus,

(4) $$E\leq \epsilon \Leftrightarrow P^{RF}\leq \eta \left(t,\epsilon \right)$$

For the received power $P^{RF}$, we use the model in [23]. For a node located at distance *r* from the AP and at a normalized angle ϕ from the direction of the serving beam, the received power is

(5) $$P^{RF}=\frac{P_{0}{\left|g\right|}^{2}F_{M}\left(\phi \right)}{1+r^{a}}$$

where $P_{0}$ is the BS transmit power, α is the path loss exponent, and *g* is the complex channel gain between the sensor node and the AP, modeled as complex Gaussian with zero mean and unit variance, i.e., g∼CN(0,1). The function $F_{M}\left(\phi \right)$ represents the Fejér kernel of order *M*, that is

(6) $$F_{M}\left(\phi \right)=\frac{1}{M}\frac{\sin^{2}\left(\frac{\pi M\phi }{2}\right)}{\sin^{2}\left(\frac{\pi \phi }{2}\right)}$$

Therefore, for a harvesting period $T_{H}$, we obtain

(7) $$\begin{array}{ccc} F_{E}\left(\epsilon \right)=P\left\{E\leq \epsilon \right\} & = & P\left\{P^{RF}\leq \eta \left(T_{H},\epsilon \right)\right\}=P\left\{{\left|g\right|}^{2}\leq \frac{\eta \left(T_{H},\epsilon \right)\left(1+r^{a}\right)}{P_{0}F_{M}\left(\phi \right)}\right\}\\ & = & 1-\exp\left(-\eta \left(T_{H},\epsilon \right)\frac{1+r^{a}}{P_{0}F_{M}\left(\phi \right)}\right) \end{array}$$

where the equality in the second line of equations is due to the fact that ${\left|g\right|}^{2}$ is exponentially distributed. For a given *r* and ϕ the average harvested energy per time slot can be found by

(8) $$E_{av,s}\left(r,\phi \right)=\int_{0}^{\infty }\left(1-F_{E}\left(\epsilon \right)\right)d\epsilon =\int_{0}^{\gamma T_{H}E_{b}}\left(1-F_{E}\left(\epsilon \right)\right)d\epsilon$$

**Figure 3:**
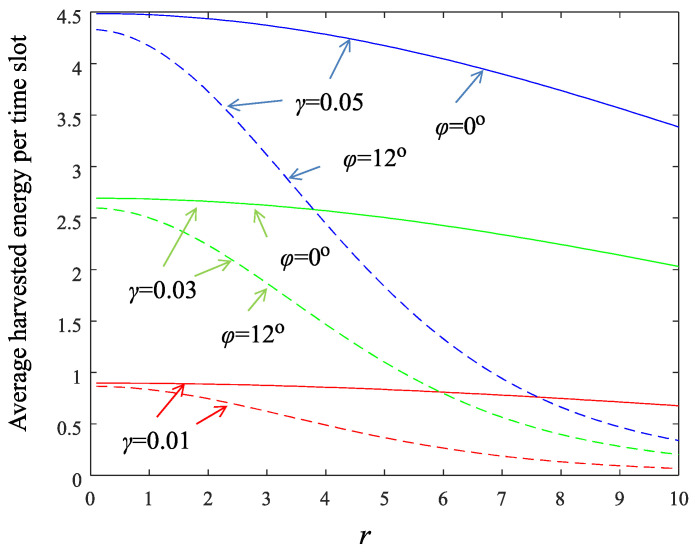
Average harvested energy $E_{av,s}\left(r,\phi \right)$ per time slot. Solid lines: $\phi =0^{\circ }$, dotted lines: $\phi =12^{\circ }$. Blue color corresponds to γ=0.05, green color to γ=0.03, and red color to γ=0.01.

Figure 3 shows the average harvested energy per slot for the parameter set used in the simulation section (see Table 1) and for three different values of the parameter γ. Note that for large distances from the AP, the harvested energy for nodes located at the edge of the beam (dotted lines, $\phi =12^{\circ }$) is considerably lower than the energy harvested by nodes located at the axial direction of the beam (solid lines, $\phi =0^{\circ }$). If a sensor node is aware of its location, i.e., explicit deployment of nodes, it can anticipate the number of time slots needed to reach a specific energy level and thus act appropriately in a case of an event. For example, for γ=0.03 and a node located at the axial direction of the beam, 50 time slots are required at most for the node to become fully charged.

If the location (r,ϕ) of the sensor node is unknown, we have to average over the spatial distribution of distances and angles in the serving sector to estimate the average harvested energy per time slot. In this case

(9) $$E_{av,s}=\frac{1}{\theta \rho^{2}}\int_{-\theta }^{\theta }\int_{0}^{\rho }\int_{0}^{\gamma T_{H}E_{b}}\exp\left(-\eta \left(T_{H},\epsilon \right)\frac{\left(1+r^{a}\right)}{P_{0}F_{M}\left(\phi \right)}\right)rdrd\phi d\epsilon$$

**Figure 4:**
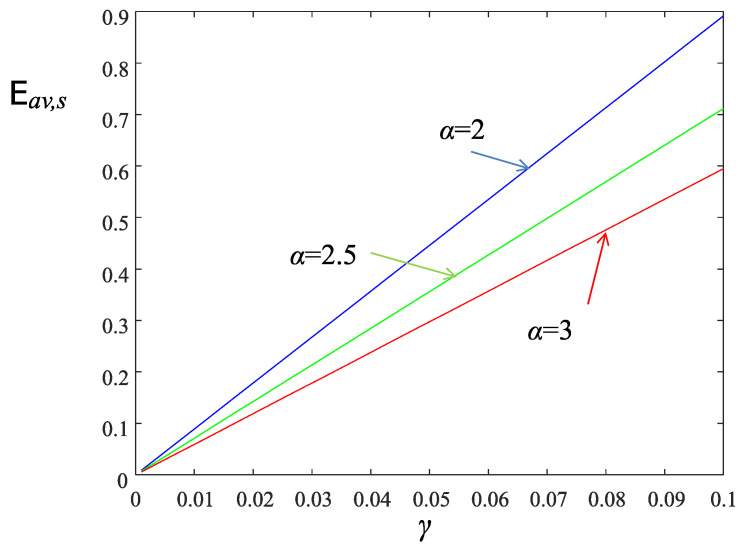
Average harvested energy $E_{av,s}$ vs. γ for various values of the path loss exponent α. Blue color corresponds to α=2, green color to α=2.5, and red color to α=3.

The average harvested energy per slot, $E_{av,s}$, is plotted vs. γ in Figure 4 for three values of the path loss exponent α. As it is expected, as α increases the amount of energy harvested by a sensor node at each time slot is reduced. The differences are magnified for larger values of the parameter γ.

### 3.3. Information Transmission Phase

Upon detection of an event, the sensing node awaits until the AP switches the beam towards its direction, charges for a period $T_{H}$ and then transmits during the period $T_{I}$.

If after charging the node’s battery is at energy level E, the transmission power is

(10) $$P=\frac{E}{T_{I}}$$

The signal-to-noise ratio (SNR) at the AP from the transmitted node located at (r,ϕ) is

(11) $$SNR\left(r,\phi \right)=\frac{{P|g|}^{2}F_{M}\left(\phi \right)}{\left(1+r^{a}\right)\sigma^{2}}$$

where $\sigma^{2}$ is the noise power. Therefore,

(12) $$\begin{array}{ccc} P\{SNR(r,\phi )\leq x\;|\;P\} & = & P\left\{\frac{{P|g|}^{2}F_{M}\left(\phi \right)}{\left(1+r^{a}\right)\sigma^{2}}\leq x\;|\;P\right\}\\ & = & P\left\{{\left|g\right|}^{2}\leq \frac{x\left(1+r^{a}\right)\sigma^{2}}{PF_{M}\left(\phi \right)}\;|\;P\right\}\\ & = & 1-\exp\left(-x\frac{\left(1+r^{a}\right)\sigma^{2}}{PF_{M}\left(\phi \right)}\right) \end{array}$$

Successful reception of the information message is possible if

(13) $$R_{I}<W^{\prime }\log_{2}\left(1+SNR\left(r,\phi \right)\right)$$

where $R_{I}$ is the information rate and $W^{\prime }$ the bandwidth allocated to a node. The information rate $R_{I}$ equals $D_{I}/T_{I}$, where $D_{I}$ is the amount of data (bits) that the node sends to the AP and $T_{I}$ is the transmission time interval ($T_{I}=T_{S}-T_{H}$). Using orthogonal signaling we assume that the available bandwidth *W* is equally divided to all nodes, and therefore

(14) $$W^{\prime }=\frac{W}{S_{u}}$$

with $S_{u}$ an upper bound on the total number of nodes. Solving Equation (13) for SNR(r,ϕ) and using Equation (12), we obtain

(15) $$\begin{array}{ccc} p_{s}\left(r,\phi \right) & = & P\{successful\;information\;reception\;for\;a\;node\;located\;at\;(r,\phi )\}\\ & = & \exp\left(-\left(2^{R_{I}S_{u}/W}-1\right)\frac{\left(1+r^{a}\right)\sigma^{2}}{PF_{M}\left(\phi \right)}\right) \end{array}$$

Again, if the location of the sensor node is unknown we integrate over the spatial distribution of the nodes to find the average probability of successful information reception.

(16) $$p_{s}=E_{\Phi }\left[p_{s}\left(r,\phi \right)\right]=\frac{1}{\theta \rho^{2}}\int_{-\theta }^{\theta }\int_{0}^{\rho }\exp\left(-\left(2^{R_{I}S_{u}/W}-1\right)\frac{\left(1+r^{a}\right)\sigma^{2}}{PF_{M}\left(\phi \right)}\right)rdrd\phi$$

**Figure 5:**
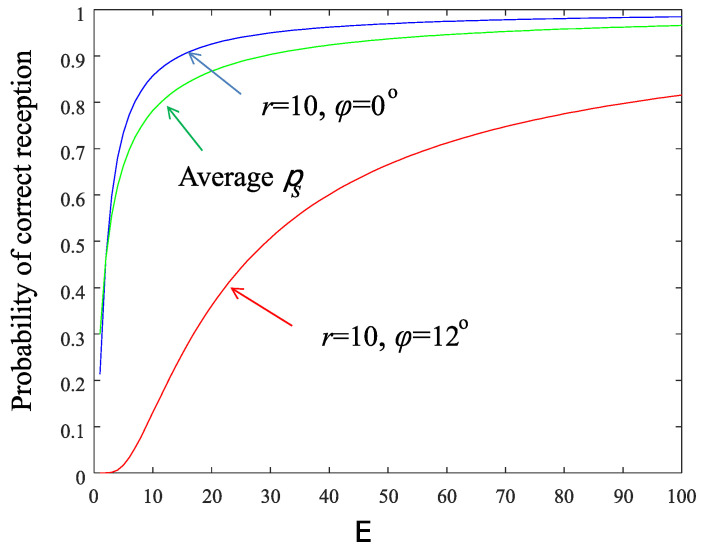
Probability of successful information reception. Green color: the average $p_{s}$, blue color: node located at $\left(10,0^{\circ }\right)$, red color: node located at $\left(10,12^{\circ }\right)$.

Figure 5 depicts the probability of successful reception of messages vs. the available energy level for two nodes located at the edge of the network (r=10). The first node is located at the axial direction of the beam (blue color) whereas the second node is located at the boundary of the sector (red color). The values of the parameters used are given in Table 1. Moreover, in Figure 5 the average probability of successful reception $p_{s}$ (Equation (16)) is plotted with green solid line.

### 3.4. Sensing Model

**Figure 6:**
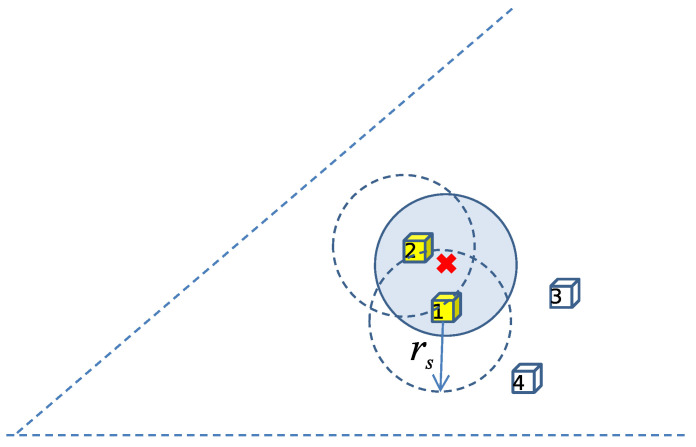
Nodes within a distance $r_{s}$ from the event (like nodes #1 and #2) can sense it.

A very simple sensing model is adopted for this work. A sensor node senses all events within a distance $r_{s}$ from its location. The situation is shown schematically in Figure 6 where nodes #1 and #2 detect the event while nodes #3 and #4 fail to do so. More sophisticated models, like the one in [24], can be used but their added value for this work is limited.

A distribution $f_{0}\left(\cdot \right)$ is used to model sensor measurements prior to the event. The same distribution, but with different parameters, say $f_{1}\left(\cdot \right)$, is used to model measurements after the event. For the rest of this paper, $f_{0}\left(\cdot \right)$ and $f_{1}\left(\cdot \right)$ are assumed to be Gaussian with means 0 and 1 respectively and standard deviation equal to 0.4. The large value of the standard deviation, compared to the difference of the means, indicates extremely noisy measurements.

## 4. Detection of Events and Data Fusion

A well-known optimal stopping technique due to Shiryayev [4] is used for event detection and described in Section 4.1. As the involved posterior probabilities are highly noisy, a Beta Particle Filter is introduced in Section 4.2 to smooth them. Finally, data fusion principles that could improve the system’s performance are presented in Section 4.3.

### 4.1. Event Detection

In the problem considered we assume for simplicity that the time at which an event occurs, T, follows the geometric distribution with probability of success ϱ. The parameter ϱ is assumed known, for example from historical data. We furthermore denote the prior probability that an event happened before starting time by π. That is

(17) $$\begin{array}{ccc} P\{T=0\} & = & \pi \end{array}$$

(18) $$\begin{array}{ccc} P\{T=n\} & = & \left(1-\pi \right){\left(1-\varrho \right)}^{n-1}\varrho \end{array}$$

The time instant *n* represents the *n*th period of beamforming the tagged sensor node. Let τ be the time at which the sensor node decides to transmit its information (measurements) to the BS. There are three risk functions associated with τ. First is the risk function $J_{1}\left(\tau \right)=P\left\{\tau <T\right\}$ which is interpreted as the probability of false alarm. Second is the risk function $J_{2}\left(\tau \right)=E\left[\max\left(\tau -T,0\right)\right]$ which is the average delay of transmission after occurrence of the event. Third is the risk function $J_{3}\left(\tau \right)=P\left\{unsuccessful\;transmission\;at\;\tau \right\}$. Minimizing the aforementioned risks has adversary effects on the selection of τ. For example, to maximize the probability of a successful transmission we have to postpone it until the sensor node is sufficiently charged to overcome bad channel conditions or even bad location conditions (distant node or edge beamforming node). This postponement imposes in turn a large delay on information reporting to the BS. If the nodes act alone, that is they do not relay information by other nodes, then it is crucial to transmit with as much power as possible in order to increase the probability the successful information reception by the BS. Thus, we formulate the problem as

(19) $$\begin{array}{ccc} & & \min P\left\{\tau <T\right\}+\lambda_{d}E\left[\max\left(\tau -T,0\right)\right]\\ & & s.t.c.\;\;P_{ui}<\psi \end{array}$$

where $P_{ui}$ denotes the probability of unsuccessful information reception and $\lambda_{d}$ is a weight factor balancing the importance between false alarm rate and detection delay. Let us first focus on the constraint and reveal the role of τ in it. Successful reception of the information message is possible if Equation (13) is satisfied. Using Equation (15) and defining for notation simplicity

(20) $$Q\left(r,\phi \right)=\frac{\left(2^{R_{I}/W^{\prime }}-1\right)\left(1+r^{a}\right)\sigma^{2}}{F_{M}\left(\phi \right)}$$

we obtain

(21) $$P_{ui}<\psi \Leftrightarrow 1-\exp\left\{-\frac{Q(r,\phi )}{P^{\tau }}\right\}<\psi \Leftrightarrow P^{\tau }>-\frac{Q(r,\phi )}{\ln(1-\psi )}$$

where $P^{\tau }$ is the transmission power at time instant τ. Moreover, $P^{\tau }$ is equal to $E^{\tau }/T_{I}$, where $E^{\tau }$ is the total energy harvested by the node up to time τ and $T_{I}$ is the signaling period. Thus, the constraint of the optimization problem (19) imposes a lower bound on the selected time $\tau^{\prime }$ to transmit. The node has to postpone its transmission at least until the accumulated stored energy satisfies

(22) $$E^{\tau^{\prime }}>-\frac{T_{I}Q\left(r,\phi \right)}{\ln(1-\psi )}$$

Inequality (22) presupposes knowledge of the location of the node. If this is not possible, the threshold on the right side of (22) may be replaced by an average over the possible locations of the sensor nodes, that is

(23) $$E^{\tau^{\prime }}>\frac{1}{\theta \rho^{2}}\int_{-\theta }^{\theta }\int_{0}^{\rho }-\frac{T_{I}Q\left(r,\phi \right)}{\ln(1-\psi )}rdrd\theta$$

We turn now to the minimization of the objective function in (19). To this end we define the posteriori probability of the event occurring before time *n*

(24) $$\pi_{n}=P\left\{T\leq n|F_{n}\right\},\;\pi_{0}=\pi$$

where $F_{n}=\sigma \left\{X_{1},X_{2},\ldots ,X_{n}\right\}$ is the σ-algebra characterizing the information before time *n*. Sensor measurements $X_{1},X_{2},\ldots$ are assumed independent having pdf $f_{0}\left(\cdot \right)$ prior to the event and pdf $f_{1}\left(\cdot \right)$ after occurrence of the event. Using Bayes’ formula

(25) $$\pi_{n+1}=\frac{\left(\pi_{n}+\left(1-\pi_{n}\right)\varrho \right)f_{1}\left(X_{n+1}\right)}{\left(\pi_{n}+\left(1-\pi_{n}\right)\varrho \right)f_{1}\left(X_{n+1}\right)+\left(1-\pi_{n}\right)\left(1-\varrho \right)f_{0}\left(X_{n+1}\right)}$$

Noting that

(26) $$P\left\{\tau <T\right\}=E\left[1-\pi_{\tau }\right]$$

and following Shiryayev, the objective function in the optimization problem (19) can be transformed into the more convenient form

(27) $$\begin{array}{ccc} J(\tau ) & = & P\left\{\tau <T\right\}+\lambda_{d}E\left[\max\left(\tau -T,0\right)\right]\\ & = & E\left[1-\pi_{\tau }+\lambda_{d}\sum_{k=0}^{\tau -1}\pi_{k}\right] \end{array}$$

For a given prior π, we seek an optimal stopping time $\tau^{🟉}$ that satisfies $J^{🟉}\left(\tau^{🟉}\right)=\min J\left(\tau \right)$. It turns out that the optimal stopping time is independent of π and can be found by a simple thresholding rule

(28) $$\tau^{🟉}=\min\left\{n\geq 0:\pi_{n}\geq \Gamma \right\}$$

Indeed, according to the results of Shiryayev (Theorem 2.23) the minimum risk function satisfies the equations

(29)J🟉(π)=min{1−π,λdπ+E[J🟉(π1)](30)=limnQn(1−π)

where $\pi_{1}$ is the posterior probability given by (25) (one step forward after the current prior π) and the expectation is over the pdf

(31) $$f\left(x\right)=\left(\pi_{n}+\left(1-\pi_{n}\right)\varrho \right)f_{1}\left(x\right)+\left(1-\pi_{n}\right)\left(1-\varrho \right)f_{0}\left(x\right)$$

The operator Q(·) is defined as

(32) $$Q\left(h\left(\pi \right)\right)=\min\{h\left(\pi \right),\lambda_{d}\pi +E\left[h\left(\pi_{1}\right)\right]\}$$

The Q(·) function is concave as the pointwise minimum of concave functions. Repeating this argument, $Q^{n}\left(1-\pi \right)$ is concave and therefore $J^{🟉}\left(\pi \right)$ is concave. The optimal stopping time $\tau^{🟉}$ is given by

(33) $$\tau^{🟉}=\min\left\{n\geq 0:\;J^{🟉}\left(\pi_{n}\right)=1-\pi_{n}\right\}$$

and due to the concavity of the risk function it assumes the form given by (19). The threshold Γ can be found using the equation

(34) $$1-\Gamma =\lambda_{d}\Gamma +J^{🟉}\left(\Gamma \right)$$

**Figure 7:**
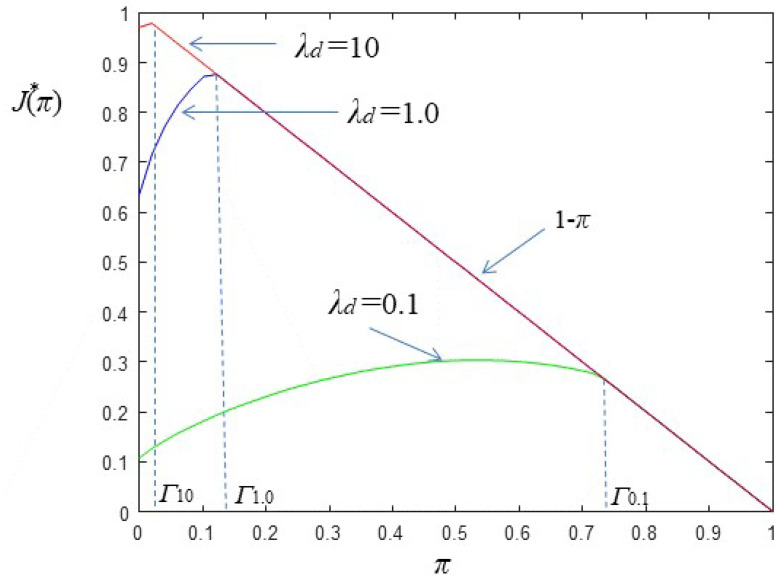
$J^{🟉}\left(\pi \right)$ and related thresholds.

Figure 7 depicts $J^{🟉}\left(\pi \right)$ for three values of $\lambda_{d}$. As $\lambda_{d}$ decreases more emphasis is given in minimizing the false alarm rate and therefore the threshold Γ drifts to larger values.

It is clear at this point that the choice of τ (the time at which the sensor node decides to transmit its information) is affected by two independent criteria. The first one is the energy criterion (22) which depends on the location of the sensor node, the probability of successful transmission 1−ψ, and the charging parameter γ. The second criterion, say false-alarm/delay criterion, is described by (28) and it depends, among other things, on $\lambda_{d}$ and the “frequency” parameter ϱ. Combining these two criteria into one is a matter of the nature of the events and the system’s parameters. For example, for false-alarm and successful reporting critical events, both criteria should be satisfied, so we can combine them as

(35) $$\tau_{o}=\max\left\{\tau^{\prime },\tau^{🟉}\right\}$$

whereas for delay critical events we can simply set

(36) $$\tau_{o}=\tau^{🟉}$$

which means that the sensor-node transmits as soon as possible regardless of its accumulated energy. Moreover, the values of the parameters of the system play a major role in the decision process, as it is demonstrated next. Consider the aforementioned network topology with ρ=10 m, S=15, sensing area of radius $r_{s}=1$ m, and the parameter ϱ set to 0.01. This means that events happen every 100 system time slots on average, which is quite high for rare events. If the events are uniformly distributed in the network area, then a specific sensor node will be “hit” with probability $\varrho r_{s}^{2}/\rho^{2}=0.0001$, that is every 10,000 time slot on average. Setting the successful transmission constraint to 1−ψ=0.9, then approximately $E_{av,s}=30$ (eyeball Figure 5). Using the curve of the smallest γ (=0.01) in Figure 3 and for the worst case of r=10, $\phi =12^{\circ }$, we find that 30/0.1=300 time slots are needed for the node to charge at the value of 30 energy units starting from zero conditions. Since the tagged sensor is charged every S=15 time slot (round-robin beamforming), the number of system time slots that are needed for the sensor to reach its target energy level is 300×15=3500. Thus the event, with high probability, will find the node sufficiently charged for transmission. In cases like this, the constraint of the optimization problem (15) can be neglected.

### 4.2. Smoothing the Posterior Probability

Measurements of the posterior probability of the event occurring before time *n*, as they are described by Equation (25) may be highly unstable. Thus, for example, a low valued threshold as $\Gamma_{10}$ or $\Gamma_{1.0}$ in Figure 7 may be crossed several times before the occurrence of an event. This in turn will impose unnecessary sensor node transmissions which will deplete its energy reserve. Therefore, if the energy harvesting process has a low rate compared to ϱ (the events’ occurrence rate), it is highly probable that at the time of an event the sensor node will be uncharged. This implies a large delay in information reporting. To remedy this problem we resort to posterior probability smoothing and we test two techniques. The first technique is straightforward and relies on the use of an AR filter whereas the second technique is a novel one and relies on particle filter principles.

#### 4.2.1. AR Smoothing

For simplicity we consider an AR filter of order one to smooth the posterior probability $\pi_{n}$. Thus,

(37) $${\tilde{\pi }}_{n}=\alpha {\tilde{\pi }}_{n-1}+\left(1-\alpha \right)\pi_{n}$$

where α∈(0,1) determines the degree of smoothing. Using a value α, we have essentially an average over the last 1/(1−α) values of the posterior probability.

**Figure 8:**
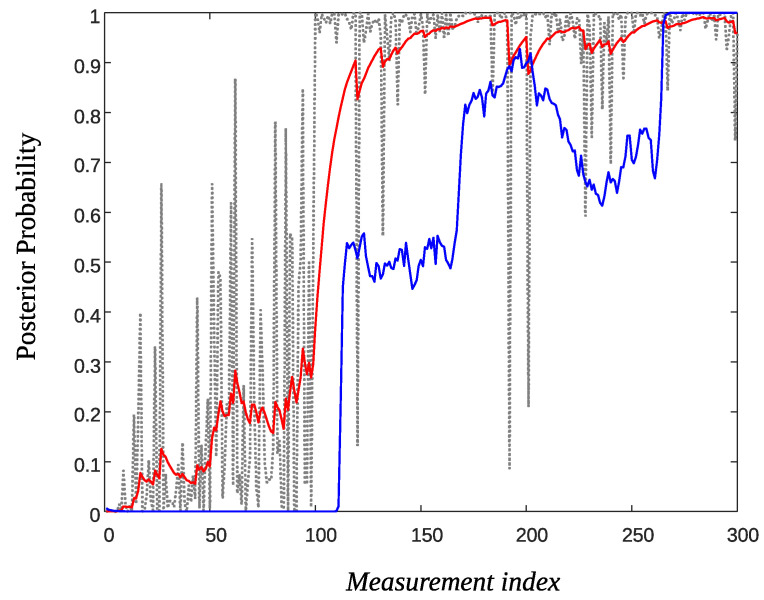
Smoothed posterior probabilities: Original $\pi_{n}$ (dotted-black), AR filter ${\tilde{\pi }}_{n}$ for α=0.9 (solid-red), and Beta Particle Filter ${\hat{\pi }}_{n}$ (solid-blue).

Figure 8 shows a trace of the posterior probability $\pi_{n}$ (Equation (25), dotted black line) generated using ϱ=0.01, $f_{0}\;∼N\left(0,0.4^{2}\right)$ and $f_{1}\;∼N\left(1,0.4^{2}\right)$. The time of the event was taken at T=100. As it is observed there are large false spikes both prior to the event and after. The red solid line depicts ${\tilde{\pi }}_{n}$ for α=0.9. Although there is some sort of smoothing there are substantial high values of the posterior probability prior to the event which may lead to several false detections. Decreasing α improves smoothing but the slope of the curve after the event decreases. This means that an extra delay may be introduced before detecting the event.

#### 4.2.2. Beta Particle Filter Smoothing

The second technique relies on the use of particle filtering and specifically the sampling-importance-resampling (SIR) method. In Bayesian filtering, such as Kalman or particle filtering, estimating the system state $x_{n}$ based on observations up to the time *n*, $y_{1:n}$, involves two steps. A prediction step to compute the prior $p(x_{n}|y_{1:n-1})$, followed by a filtering step to compute the posterior $p(x_{n}|y_{1:n})$. Thus

(38) $$\begin{array}{ccc} Prediction: & & p\left(x_{n}|y_{1:n-1}\right)=\int p\left(x_{n}|x_{n-1}\right)p\left(x_{n-1}|y_{1:n-1}\right)dx_{n-1} \end{array}$$

(39) $$\begin{array}{ccc} Filtering: & & p\left(x_{n}|y_{1:n}\right)\propto \int p\left(y_{n}|x_{n}\right)p\left(x_{n}|y_{1:n-1}\right) \end{array}$$

In the proposed smoothing technique, the posterior probabilities $\pi_{n}$, given by Equation (25), play the role of the observations $y_{n}$ while the smoothed values ${\hat{\pi }}_{n}$ will represent the unknown internal system state $x_{n}$. In both the prediction step and filtering step we use the Beta distribution to model the state transition probability distribution $p\left(x_{n}|x_{n-1}\right)=p\left({\hat{\pi }}_{n}|{\hat{\pi }}_{n-1}\right)$ and the likelihood $p\left(y_{n}|x_{n}\right)=p\left(\pi_{n}|{\hat{\pi }}_{n}\right)$. The Beta distribution is given by

(40) $${Beta}_{x}\left(a,b\right)=\frac{\Gamma (a+b)}{\Gamma (a)\Gamma (b)}x^{a-1}{\left(1-x\right)}^{b-1}$$

with the shape parameters *a* and *b* controlling the peak and the sharpness of the peak and Γ(·) is the Gamma function. Since the filtered value ${\hat{\pi }}_{n}$ has to be close to ${\hat{\pi }}_{n-1}$ we model the state transition probability as

(41) $$p\left({\hat{\pi }}_{n}|{\hat{\pi }}_{n-1}\right)∼{Beta}_{{\hat{\pi }}_{n}}\left(\nu_{1}{\hat{\pi }}_{n-1},\nu_{1}\left(1-{\hat{\pi }}_{n-1}\right)\right)$$

with the parameter $\nu_{1}$ taking a large value, to guarantee a>1 and b>1. Similarly, we model the likelihood as

(42) $$p\left(\pi_{n}|{\hat{\pi }}_{n}\right)∼{Beta}_{\pi_{n}}\left(\nu_{2}{\hat{\pi }}_{n}+1,\nu_{2}\left(1-{\hat{\pi }}_{n}\right)+1\right)$$

with the parameter $\nu_{2}$ having a relatively small value. The bias term (+1) was introduced to avoid numerical problems when $\pi_{n}$ assumes small values. The Beta Particle Filtering algorithm is presented in Algorithm 1.
**Algorithm 1** The Beta Particle Filter smoothing algorithm.**Initialization**         • Select the number of particle streams *K*         • Generate *K* samples for the initial state ${\hat{\pi }}_{0}=\pi$          **For** k=1,…,K             - draw ${\hat{\pi }}_{0}^{k}∼{Beta}_{{\hat{\pi }}_{0}}\left(\nu_{1}\pi ,\nu_{1}\left(1-\pi \right)\right)$             - set $w_{0}^{k}=1/K$ % set initial weights          **end for****Main Loop**         **For** n=1,2,…          **For** k=1,2,…,K             - draw ${\hat{\pi }}_{n}^{k}∼{Beta}_{{\hat{\pi }}_{n}}\left(\nu_{1}{\hat{\pi }}_{n-1}^{k},\nu_{1}\left(1-{\hat{\pi }}_{n-1}^{k}\right)\right)$             - $w_{n}^{k}=w_{n-1}^{k}{Beta}_{\pi_{n}}\left(\nu_{2}{\hat{\pi }}_{n}^{k}+1,\nu_{2}\left(1-{\hat{\pi }}_{n}^{k}\right)+1\right)$          **end for**          $W_{n}^{k}=w_{n}^{k}/\sum_{k}w_{n}^{k}$ % normalize weights          $K_{eff}=1/\sum_{k}{\left(W_{n}^{k}\right)}^{2}$          **If** $K_{eff}<K_{T}$ % $K_{T}$ was taken equal to 0.85K             - Resample ${\left\{{\hat{\pi }}_{n}^{k},W_{n}^{k}\right\}}_{k=1}^{K}$ to obtain ${\left\{{\bar{\pi }}_{n}^{k},1/K\right\}}_{k=1}^{K}$             - Set ${\hat{\pi }}_{n}^{k}={\bar{\pi }}_{n}^{k}$, $W_{n}^{k}=1/K$          **end if**          • Estimate ${\hat{\pi }}_{n}=\sum_{k}{\hat{\pi }}_{n}^{k}W_{n}^{k}$         **end for**

The solid-blue line in Figure 8 depicts the smoothed value ${\hat{\pi }}_{n}$ using the Beta Particle Filter method. The plot was obtained using K=200 particle streams and the parameters $\left(\nu_{1},\nu_{2}\right)=\left(500,1\right)$. As it is observed, prior to the event the smoothed posterior probability stays close to zero but there is a substantial delay after the event to reach the true value one.

### 4.3. Fusion of the Sensor Measurements

From the discussion in the previous subsection, it becomes clear that more reliable estimates of the posterior probability are needed before the sensor nodes decide to transmit their information. This may be achieved by fusing the measurements of the sensors that cover the same area. To this end, sensor nodes utilize the second time-slot format and exchange measurement information during $T_{F}$ prior to any transmission.

**Figure 9:**
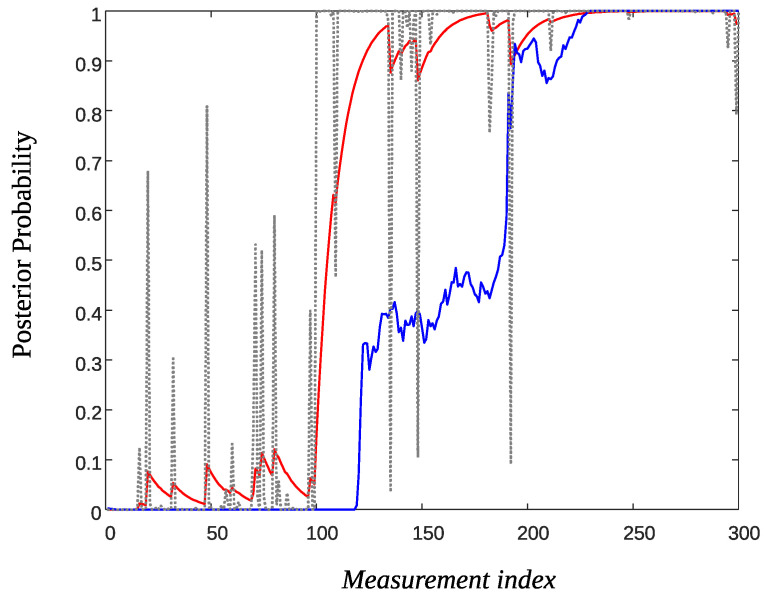
Data fusion of three sensors for smoothing the posterior probabilities: Original $\pi_{n}$ (dotted-black), AR filter ${\tilde{\pi }}_{n}$ for α=0.9 (solid-red) and Beta Particle Filter ${\hat{\pi }}_{n}$ (solid-blue).

Figure 9 shows the smoothed posterior probabilities of three sensors and as it is observed false alarm probabilities (prior to the event and for the AR filtering technique), have been reduced by a factor 2 to 3 compared to that of Figure 8. Note that if *m* sensors are involved in the fusion process, the posterior probabilities (Equation (25)) are given by

(43) $$\pi_{n+1}=\frac{\left(\pi_{n}+\left(1-\pi_{n}\right)\rho \right)\prod_{i=1}^{m}f_{1}^{\left(i\right)}\left(X_{n+1}^{\left(i\right)}\right)}{\left(\pi_{n}+\left(1-\pi_{n}\right)\rho \right)\prod_{i=1}^{m}f_{1}^{\left(i\right)}\left(X_{n+1}^{\left(i\right)}\right)+\left(1-\pi_{n}\right)\left(1-\rho \right)\prod_{i=1}^{m}f_{0}^{\left(i\right)}\left(X_{n+1}^{\left(i\right)}\right)}$$

However, there is a price to pay for the advantages of fusion. Covering an area of interest by more than one sensor node is not always feasible and it depends mainly on the type of sensors used. For example, an optical sensor has a much larger sensing radius compared to a temperature sensor but nevertheless, line-of-sight blocking limits the size of the monitored area. Let us assume that the distribution of the sensor nodes is a homogeneous Poisson Point Process (PPP) with intensity λ. If it is desirable for the event to be detected by at least two sensors with a probability higher than $\alpha_{c}$, then

(44) $$P\left\{\#sensors\geq 2\right\}\geq \alpha_{c}\Rightarrow 1-\left(e^{-\lambda A_{s}}+e^{-\lambda A_{s}}\lambda A_{s}\right)\geq \alpha_{c}$$

with $A_{s}=\pi r_{s}^{2}$. Setting $r_{s}=1$ m and $\alpha_{s}=0.5$ results in 168 nodes on average that have to be deployed over an area of radius 10 m. If the sensing radius is reduced to 0.5 m then the number of nodes has to be quadrupled. Moreover, using an extra time period $T_{F}$ in the time slot format in order for the sensors to exchange their measurements, reduces either the harvesting period $T_{H}$, or the transmission period $T_{I}$, or both. One may neglect $T_{F}$ and resort to centralized information fusion by letting all the sensors sensing an event independently transmit their measurements to the AP.

There are also certain advantages to introducing the time period $T_{F}$. During this period sensor nodes may exchange information, beyond measurements for data fusion, such as their energy reserves. In this case, more sophisticated transmission strategies may be explored. For example, among two or more sensor nodes that detect an event, only the node with the higher energy level transmits the information to the AP. The rest of the nodes delay their transmission either for extra charging purposes or to increase time diversity. Since for neighboring nodes the channel gains are highly correlated, postponing the transmission for a period greater than the time coherence of the channel may prevent nodes transmitting under the same (possibly bad) channel conditions.

## 5. Simulation Results

In this section, some simulation results are presented that corroborate the analysis of the previous section. The values of the system parameters are given in Table 1 taken from [6] and the simulation platform was MATLAB.

In our simulation model, a base station lies at the center of a cell of radius 10 m and a linear antenna array of M = 8 elements is used to energy beamform 15 sectors in a round-robin fashion. The location of the event is selected randomly in the monitored area and the event interarrival time follows the geometric distribution. Channel gains are modeled as independent random variables following an exponential distribution. We assume fast varying channels in the sense that the gains change at every time slot.

We focus on the behavior of two sensor nodes located at the edge of the network, that is at a distance r=10 m from the AP. The first node lies along the direction of the beam pattern ($\phi =0^{\circ }$), whereas the second sensor node lies at $\phi =12^{\circ }$, which is at the edge of the beam pattern. Sensor information transmission and charging are possible only when the AP beamforms towards the tagged sector. However, the nodes continuously sense the environment for possible events and run the detection algorithm. The simulation horizon is large enough to ensure the creation of several events in the neighborhood of the sensors.

We present two sets of simulations. In the first set we illustrate the effects of smoothing, charging efficiency γ, and event probability ϱ on the false alarm rate and the accumulated node energy prior to information transmission. In the second set, we deal with the effects of the aforementioned parameters on the information reporting delay.

### 5.1. Charging and False Alarm Rate

**Figure 10:**
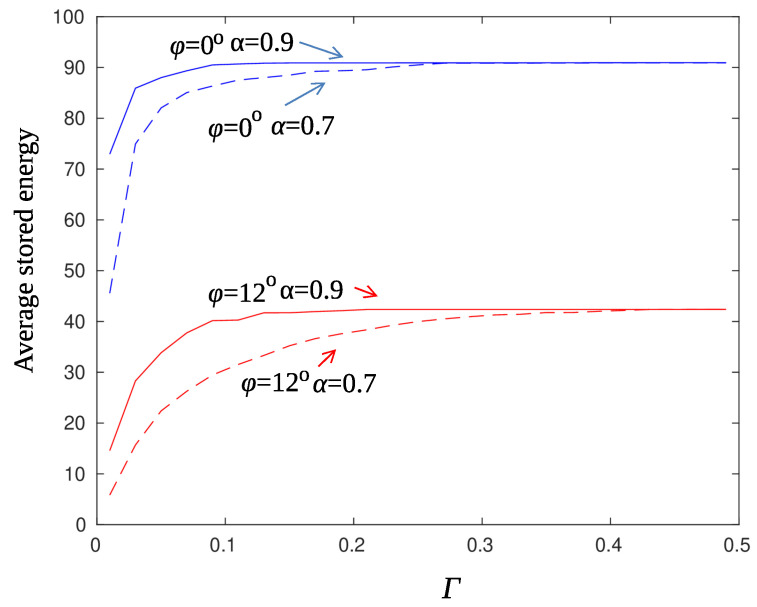
AR filter—average stored energy for γ=0.01, ϱ=0.1.

**Figure 11:**
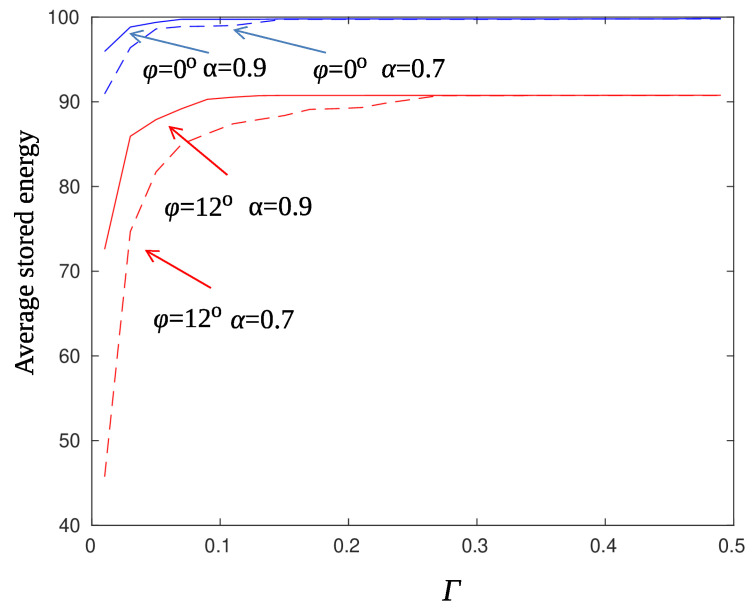
AR filter—average stored energy for γ=0.1, ϱ=0.1.

Figure 10 shows the average accumulated energy for various values of the threshold parameter Γ (Criterion 36). The averages were obtained using 200 runs, the charging efficiency was set to γ=0.01 and AR smoothing of the posterior probabilities was used. A few observations are immediate. First of all, the node at the edge of the beam pattern fails to reach high energy levels as it is with the node along the main direction of the beam. A remedy to this problem is to offset the direction of the beam (by half of the beam width) before starting a new charging cycle. Secondly, the energy storage from one value onwards remains constant. Values of the threshold Γ greater than 0.3 do not affect the charge percentage of the sensors. For small values of Γ, the threshold is exceeded several times forcing the sensor to transmit and therefore to deplete regularly its energy storage. Figure 11 repeats the experiment with a larger value of the charging efficiency parameter γ. In this case, sensors charge at a tenfold rate and manage to recover from false transmissions.

The effect of smoothing the posterior probabilities is more obvious for small values of the threshold Γ. As it is observed from Figure 10 and Figure 11, high smoothing (α=0.9) prevents, to some extent, false alarms and moderates unnecessary threshold crossings.

**Figure 12:**
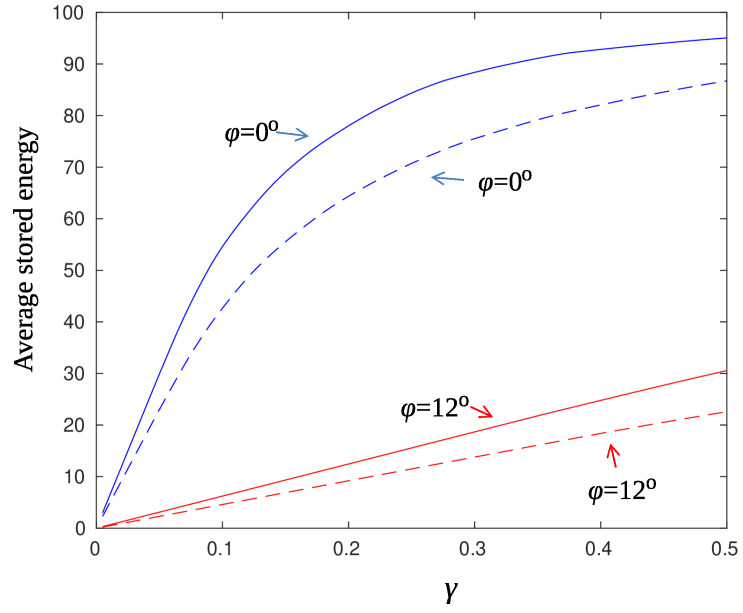
Average stored energy for Γ=0.15, ϱ=1, α=0.9, and r=10 m. Solid line: Particle filter method, dashed line: AR filtering.

Figure 12 compares the performance of the Beta Particle Filter smoother with that of the AR filter using α=0.9. The threshold Γ was set equal to 0.15 and the accumulated energy prior to information transmission is plotted vs. the charging efficiency γ. Note that for this experiment ϱ=1, which means a high rate of events. One event occurs in the monitored network area every one time slot on average. It is conceivable from the figure that BPF outperforms AR smoothing by almost 10%.

**Figure 13:**
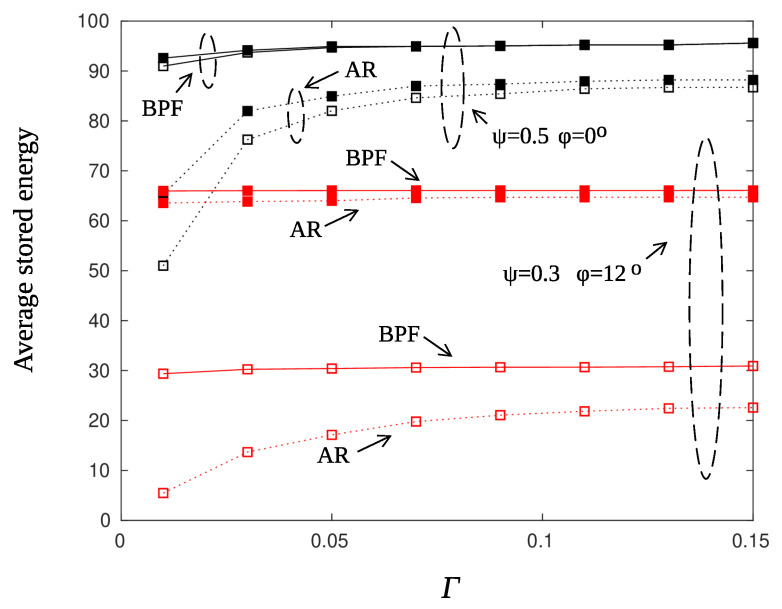
Average stored energy for particle filter (solid line) and AR filter (dotted line), with parameters γ=0.5, ϱ=1, r=10 m, ψ=0.05, $\phi =0^{\circ }$ (black lines), and ψ=0.3, $\phi =12^{\circ }$ (red lines). Filled squares mark both threshold and energy criteria (Cr. 35), squares mark only threshold criteria (Cr. 36).

Figure 13 compares the performance of the smoothing filters under detection criteria 35 and 36. Black lines correspond to the case of a sensor that lies on the main direction of the beam pattern $\phi =0^{\circ }$. The target probability of unsuccessful transmission was set equal to ψ=0.05. There are two observations that reveal the superiority of the BPF against AR smoothing regarding the treatment of false alarms. Firstly, using BPF the sensor node reaches higher values of accumulated energy that are almost independent on the threshold parameter Γ. Secondly, Criterion 35, that is postponement of information transmission until both energy and detection threshold constraints are satisfied, does not have a major impact on the accumulated energy. This is justified by the fact that the BPF smoother is not “fooled” by temporary ripples of the posterior probabilities and therefore the sensor node does not discharge for unnecessary transmissions. Red lines correspond to the case of a sensor that lies at the edge of the beam pattern $\phi =12^{\circ }$. Although the BPF smoother has steadily better performance than the AR filter, the differences are not that large. An edge sensor is hard to charge and therefore it is difficult to recover after false alarms. In cases like this, it is preferable to postpone transmissions until the sensor is sufficiently charged in order to guarantee the successful transmission of the information message.

### 5.2. Charging and Detection Delay

**Figure 14:**
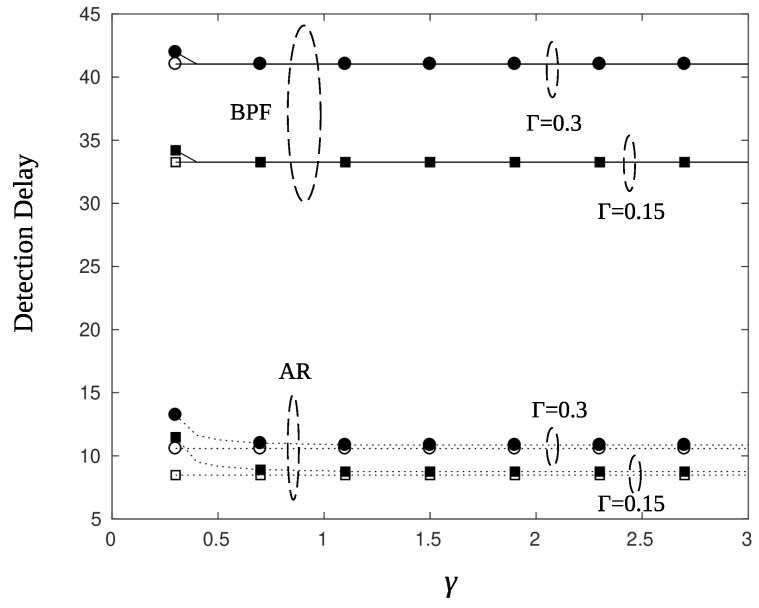
Detection delay for particle filter (solid line) and AR filter (dotted line), with parameters $\phi =0^{\circ }$, ϱ=1, ψ=0.05, r=10 m, Γ=0.15 (square), and Γ=0.3 (circle). Filled squares mark both threshold and energy criteria (Cr. 35), squares mark only threshold criteria (Cr. 36).

**Figure 15:**
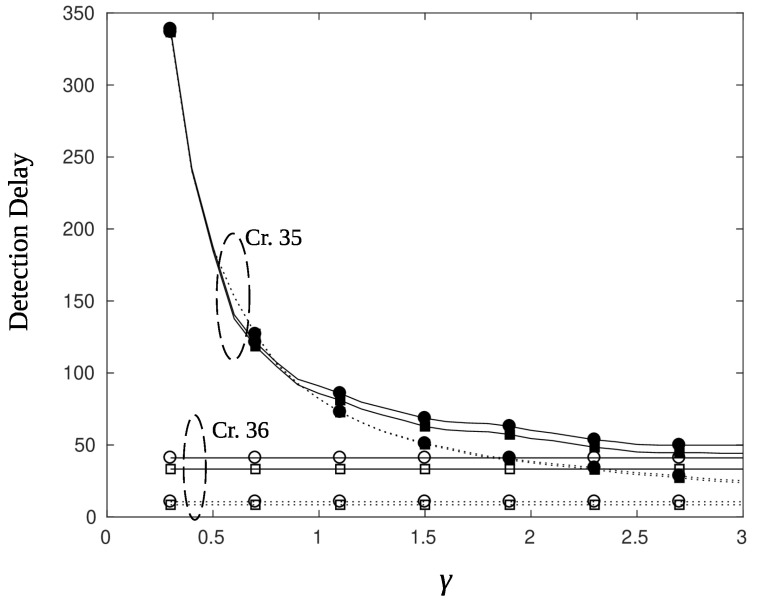
Detection delay for particle filter (solid line) and AR filter (dotted line), with parameters $\phi =12^{\circ }$, ϱ=1, ψ=0.3, r=10 m, Γ=0.15 (square), and Γ=0.3 (circle). Filled squares mark both threshold and energy criteria (Cr. 35), squares mark only threshold criteria (Cr. 36).

Figure 14 plots the detection delay, measured in time slots, for a sensor that charges well ($\phi =0^{\circ }$ and high values of the charging efficiency parameter γ. An immediate observation is that the use of BPF results in larger delays compared to that of AR filter by almost 25–30 time slots. This is in agreement with the results of Figure 8 and Figure 9, where it is demonstrated that the response of the BPF to the changes of the posterior likelihood is delayed. Another, rather obvious, observation is that smaller values of threshold parameter Γ produces smaller delays. Indeed, comparing the circle marked curves, which correspond to Γ=0.3, to the square marked curves, which correspond to Γ=0.15, we note a difference in the detection delay of 10 time slots for the BPF and 4 time slots for the AR filter. The criterion selection, i.e., criterion 35 or 36, does not have a major impact on the detection delay for nodes that charge easily. As it is observed from Figure 14 criterion 35 (filled square and circle markers) provides the same performance with criterion 36 (empty markers). However, this is not the case for sensor nodes that are difficult to charge, as is shown next. Figure 15, depicts the detection delay performance for a node that lies at the edge ($\phi =12^{\circ }$) of the charging beam. Unless the charging efficiency γ is high enough, detection delays reach large values if the node has to postpone its transmission (criterion 35, filled markers). Thus, in such cases alternative solutions must be sought. For example, one may take off edge sensors from their unfavorable position by beamforming using an angle offset or by letting sensor nodes relay the information of their neighbors. In the latter scheme, nodes transmit using less power since the objective is to reach a neighbor node that is well charged and capable to relay the information to the AP.

**Figure 16:**
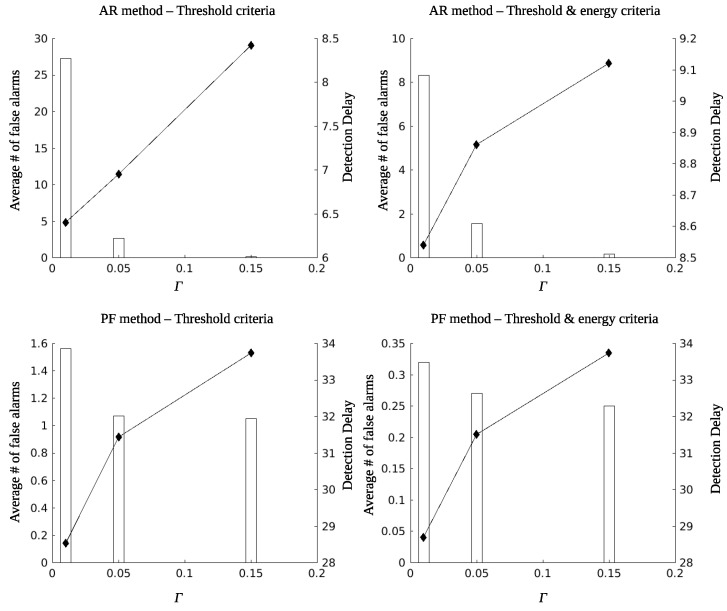
Detection delay—average number of false alarms before the event, with parameters $\phi =0^{\circ }$, ϱ=1, ψ=0.05, r=10 m, γ=0.5, and α=0.9.

Figure 16 summarizes some comparative results for the case of a sensor that charges well ($\phi =0^{\circ }$). The left column of figures corresponds to criterion 36 whereas the right column corresponds to criterion 35. The first row of figures is for AR filtering (α=0.9) and the second row is for Beta Particle Filter smoothing. The average number of false alarms is depicted with bars, whereas the detection delay is depicted with filled diamond marks. As it is observed, for very small values of the threshold parameter Γ, the Beta Particle Filter smoothing results in considerably smaller values (tenfold reduction) of false alarms compared to AR filtering. However, the situation is reversed regarding the detection delay. Using criterion 35, although the detection delay is not affected, the false alarm rates are reduced by a factor of three (or more) for small values of the parameter Γ.

## 6. Discussion

A thorough study was conducted on the performance aspects of a WPCN network used to detect events under the assumption of noisy sensor measurements. Detecting changes in the distribution of environmental parameters was based on the Optimal Stopping Theory by Shiryaev. The unstable nature of the involved likelihoods imposes smoothing on the posterior estimates and to this end, a Beta Particle Filter was proposed and tested against a classical AR filter. Analytical and simulation results show that performance indices, such as false alarm rate, detection delay, and probability of successful transmission, rely heavily on the charging efficiency and the location of the sensor nodes. Future work is oriented to incorporating mechanisms, such as data/information fusion and relaying of information, that alleviates the problem of partly charged sensor nodes, in an effort to increase the reliability of the system.

## Figures and Tables

**Table 1:** Parameters used in simulations.

Parameter	Value
$\zeta_{1}$, $\zeta_{2}$, $E_{b}$	1500, 0.0022, 100
ρ, θ, α, *M*	10 m, π/15, 2, 8
$P_{0}$, $\sigma^{2}$, λ	−10 db, −30 db, 1
$D_{I}$, $W^{\prime }$	1024 bits, 1 KHz

## Data Availability

Data set available on request to corresponding authors.

## References

[1] Djidi N.E.H., Gautier M., Courtay A., Berder O., Magno M. (2021). How can wake-up radio reduce lora downlink latency for energy harvesting sensor nodes?. Sensors.

[2] Ahmad I., Hee L.M., Abdelrhman A.M., Imam S.A., Leong M.S. (2021). Scopes, challenges and approaches of energy harvesting for wireless sensor nodes in machine condition monitoring systems: A review. Measurement.

[3] Shiryaev A.N. (1963). On optimum methods in quickest detection problems. Theory Probab. Its Appl..

[4] Shiryaev A.N. (2007). Optimal Stopping Rules.

[5] Guo J., Zhou X., Durrani S. (2018). Wireless power transfer via mmWave power beacons with directional beamforming. IEEE Wirel. Commun. Lett..

[6] Psomas C., Krikidis I. (2018). Energy beamforming in wireless powered mmWave sensor networks. IEEE J. Sel. Areas Commun..

[7] Hong Y.W.P., Hsu T.C., Chennakesavula P. (2016). Wireless power transfer for distributed estimation in wireless passive sensor networks. IEEE Trans. Signal Process..

[8] Koutsioumpos M., Zervas E., Hadjiefthymiades E., Merakos L. (2020). Monitoring for Rare Events in a Wireless Powered Communication mmWave Sensor Network. Sensors.

[9] Godala S., Vaddella R.P.V. (2020). A study on intrusion detection system in wireless sensor networks. Int. J. Commun. Netw. Inf. Secur..

[10] Kandris D., Nakas C., Vomvas D., Koulouras G. (2020). Applications of wireless sensor networks: An up-to-date survey. Appl. Syst. Innov..

[11] Safaei M., Asadi S., Driss M., Boulila W., Alsaeedi A., Chizari H., Abdullah R., Safaei M. (2020). A systematic literature review on outlier detection in wireless sensor networks. Symmetry.

[12] Tartakovsky A.G., Rozovskii B.L., Blažek R.B., Kim H. (2006). Detection of intrusions in information systems by sequential change-point methods. Stat. Methodol..

[13] Tartakovsky A.G., Veeravalli V.V. (2004). Change-point detection in multichannel and distributed systems. Appl. Seq. Methodol. Real-World Examples Data Anal..

[14] Xie Y., Siegmund D. Sequential multi-sensor change-point detection. Proceedings of the 2013 Information Theory and Applications Workshop (ITA).

[15] Raghavan V., Veeravalli V.V. (2010). Quickest change detection of a Markov process across a sensor array. IEEE Trans. Inf. Theory.

[16] Hadjiliadis O., Zhang H., Poor H.V. (2009). One shot schemes for decentralized quickest change detection. IEEE Trans. Inf. Theory.

[17] Moustakides G.V. (1986). Optimal stopping times for detecting changes in distributions. Ann. Stat..

[18] Veeravalli V.V. (2001). Decentralized quickest change detection. IEEE Trans. Inf. Theory.

[19] Premkumar K., Kumar A. Optimal sleep-wake scheduling for quickest intrusion detection using wireless sensor networks. Proceedings of the IEEE INFOCOM 2008-The 27th Conference on Computer Communications.

[20] Zervas E., Mpimpoudis A., Anagnostopoulos C., Sekkas O., Hadjiefthymiades S. (2011). Multisensor data fusion for fire detection. Inf. Fusion.

[21] Lorden G. (1971). Procedures for reacting to a change in distribution. Ann. Math. Stat..

[22] Boshkovska E., Ng D.W.K., Zlatanov N., Schober R. (2015). Practical non-linear energy harvesting model and resource allocation for SWIPT systems. IEEE Commun. Lett..

[23] Ding Z., Fan P., Poor H.V. (2017). Random beamforming in millimeter-wave NOMA networks. IEEE Access.

[24] Manatakis D.V., Manolakos E.S. (2014). Estimating the spatiotemporal evolution characteristics of diffusive hazards using wireless sensor networks. IEEE Trans. Parallel Distrib. Syst..

